# A higher non-severe hypoglycaemia rate is associated with an increased risk of subsequent severe hypoglycaemia and major adverse cardiovascular events in individuals with type 2 diabetes in the LEADER study

**DOI:** 10.1007/s00125-021-05556-7

**Published:** 2021-10-26

**Authors:** Simon R. Heller, Milan S. Geybels, Ahmed Iqbal, Lei Liu, Lily Wagner, Elaine Chow

**Affiliations:** 1 Department of Oncology and Metabolism, University of Sheffield, Sheffield, UK; 2 Novo Nordisk A/S, Søborg, Denmark; 3 Department of Infection, Immunity and Cardiovascular Disease, University of Sheffield, Sheffield, UK

**Keywords:** Cardiac complications, Hypoglycaemia, Macrovascular disease

## Abstract

**Aims/hypothesis:**

Hypoglycaemia is a common side effect of insulin and some other antihyperglycaemic agents used to treat diabetes. Severe hypoglycaemia has been associated with adverse cardiovascular events in trials of intensive glycaemic control in type 2 diabetes. The relationship between non-severe hypoglycaemic episodes (NSHEs) and severe hypoglycaemia in type 2 diabetes has been documented. However, an association between more frequent NSHEs and cardiovascular events has not been verified. This post hoc analysis of the LEADER (Liraglutide Effect and Action in Diabetes: Evaluation of Cardiovascular Outcome Results) trial aimed to confirm whether there is an association between NSHEs and severe hypoglycaemic episodes in individuals with type 2 diabetes. In addition, the possible association between NSHEs and major adverse cardiac events (MACE), cardiovascular death and all-cause mortality was investigated.

**Methods:**

LEADER was a double-blind, multicentre, placebo-controlled trial that found that liraglutide significantly reduced the risk of MACE compared with the placebo. In this post hoc analysis, we explored, in all LEADER participants, whether the annual rate of NSHEs (defined as self-measured plasma glucose <3.1 mmol/l [56 mg/dl]) was associated with time to first severe hypoglycaemic episode (defined as an episode requiring the assistance of another person), time to first MACE, time to cardiovascular death and time to all-cause mortality. Participants with <2 NSHEs per year were used as reference for HR estimates. Cox regression with a time-varying covariate was used.

**Results:**

We demonstrate that there is an association between NSHEs (2–11 NSHEs per year and ≥12 NSHEs per year) and severe hypoglycaemic episodes (unadjusted HRs 1.98 [95% CI 1.43, 2.75] and 5.01 [95% CI 2.84, 8.84], respectively), which was consistent when baseline characteristics were accounted for. Additionally, while no association was found between participants with 2–11 NSHEs per year and adverse cardiovascular outcomes, higher rates of NSHEs (≥12 episodes per year) were associated with higher risk of MACE (HR 1.50 [95% CI 1.01, 2.23]), cardiovascular death (HR 2.08 [95% CI 1.17, 3.70]) and overall death (HR 1.80 [95% CI 1.11, 2.92]).

**Conclusions/interpretation:**

The analysis of data from the LEADER trial demonstrated that higher rates of NSHEs were associated with both a higher risk of severe hypoglycaemia and adverse cardiovascular outcomes in individuals with type 2 diabetes. Therefore, irrespective of the cause of this association, it is important that individuals with high rates of hypoglycaemia are identified so that the potentially increased risk of cardiovascular events can be managed and steps can be taken to reduce NSHEs.

**Trial registration:**

ClinicalTrials.gov (NCT01179048).

**Graphical abstract:**

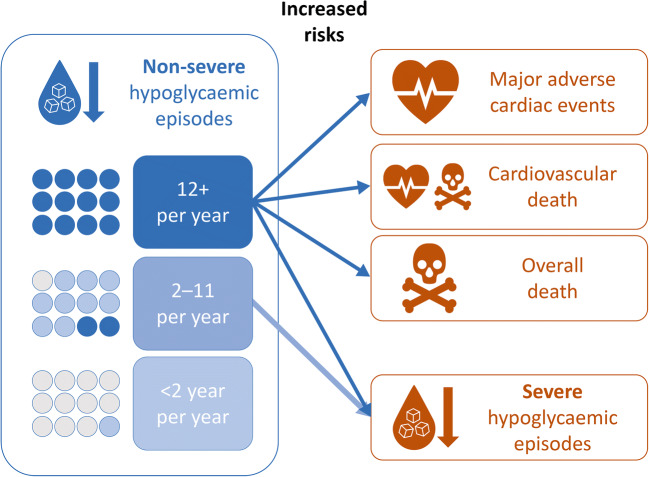

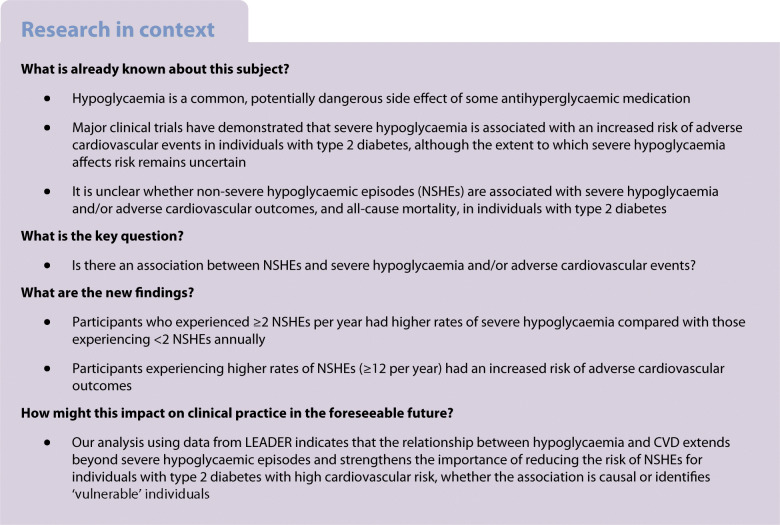



## Introduction

There is considerable evidence that individuals with type 1 and type 2 diabetes benefit greatly from maintaining good glycaemic control [1, 2]; however, many do not reach their treatment targets [3]. Unwanted treatment-associated side effects, such as hypoglycaemia, are a barrier to optimal glycaemic control in individuals with diabetes [4].

Hypoglycaemia is a common, potentially dangerous, side effect of diabetes therapy, particularly with insulins and sulfonylureas [5, 6], that may lead to confusion, coma and seizures [7, 8]; and, unsurprisingly, fear of future episodes affects both individuals and family members [9].

Analyses of major clinical trials have investigated the relationship between severe hypoglycaemia (defined as cognitive impairment requiring assistance from another person) and adverse cardiovascular outcomes. These analyses demonstrated that severe hypoglycaemia was associated with an increased risk of all-cause mortality and/or adverse cardiovascular outcomes in individuals with type 2 diabetes [10–15]. In contrast, in ACCORD (Action to Control Cardiovascular Risk in Diabetes), while an increased risk of death was seen in all participants experiencing symptomatic and severe hypoglycaemia, the risk of death was lower in those experiencing one or more hypoglycaemic episodes receiving intensive glucose control vs standard glucose control [11, 12]. This suggests that factors other than severe hypoglycaemia account for the mortality difference between the two arms. Susceptibility to severe hypoglycaemia could merely be a marker for an underlying disease that increases the mortality risk for these individuals. Alternatively, the reduced risk of mortality observed in patients treated intensively and experiencing hypoglycaemia may be due to repeated episodes of hypoglycaemia leading to impaired sympathoadrenal responses, which are linked to adverse cardiovascular effects [16].

Importantly, while an association between severe hypoglycaemia and an increased risk of adverse cardiovascular events has been demonstrated, there is also evidence that previous cardiovascular events are associated with severe hypoglycaemia [17, 18]. However, recent evidence has shown that the risk of cardiovascular events appears to increase with an increasing hypoglycaemia risk score, supporting the hypothesis that hypoglycaemia could be a risk factor for adverse cardiovascular outcomes [19]. The bi-directional relationship between severe hypoglycaemia and cardiovascular, and other non-vascular, conditions may merely reflect increased vulnerability to hypoglycaemia and these other conditions [13, 17]. If severe hypoglycaemia identifies ‘sicker individuals’ who are more likely to experience cardiovascular events, then it could be considered a risk marker of CVD, irrespective of whether a causal link exists.

Non-severe hypoglycaemic episodes (NSHEs) have been associated with a higher risk of cardiovascular events, hospitalisation and all-cause mortality in some studies of individuals with type 2 diabetes, and of critically ill individuals with and without diabetes [20, 21]. However, other studies have found no association of NSHEs with cardiovascular outcomes observed in participants with dysglycaemia and at high risk of cardiovascular events [22], or in those with type 2 diabetes when measuring cardiovascular deaths over 4 years [23].

Higher rates of NSHEs have been shown to be associated with a greater risk of severe hypoglycaemia in individuals with type 1 diabetes [24, 25], but the association in those with type 2 diabetes is unclear [22, 26, 27]. Some studies have reported an association [26, 27], but others, including the ORIGIN (Outcome Reduction With Initial Glargine Intervention) trial, found no association following adjustment for confounding variables [22]. The link between NSHEs and severe hypoglycaemia is well recognised, but the association of more frequent NSHEs with cardiovascular events remains unresolved. To explore this relationship further, we examined the frequencies of non-severe and severe hypoglycaemic events in a large population of patients with type 2 diabetes and an increased cardiovascular risk.

LEADER (Liraglutide Effect and Action in Diabetes: Evaluation of Cardiovascular Outcome Results) was a cardiovascular outcomes trial with a median follow-up of 3.8 years comparing liraglutide with placebo in addition to standard of care in individuals with type 2 diabetes and high cardiovascular risk [28]. The size and design of the LEADER trial, and the relatively large number of NSHEs (defined as an event with symptoms consistent with hypoglycaemia confirmed by a concomitant glucose reading <3.1 mmol/l [56 mg/dl]), therefore provide an opportunity to investigate the association between NSHEs and severe hypoglycaemic episodes in individuals with type 2 diabetes. This analysis also aims to investigate the possible associations between NSHEs and major adverse cardiac events (MACE), cardiovascular death and all-cause mortality, but does not aim to determine causality.

## Methods

### Study design and participants

The post hoc analyses described below utilised data from the LEADER trial (NCT01179048). Detailed descriptions of the protocol, methods and primary results have been published previously [28, 29]. In brief, LEADER was a double-blind, multicentre, placebo-controlled trial that included 9340 participants with uncontrolled type 2 diabetes (HbA_1c_ ≥ 53 mmol/mol [7%]) either ≥50 years old with pre-existing CVD (81%), or ≥60 years old with at least one cardiovascular risk factor (19%), as determined by the investigator. Participants were randomised 1:1 to receive either 1.8 mg (or the maximum tolerated dose) of liraglutide or a matching placebo once daily in addition to standard of care. Those that did not meet their target for glycaemic control (HbA_1c_ ≤ 53 mmol/mol [7%] or an individualised target) after randomisation could receive additional antihyperglycaemic agents (excluding glucagon-like peptide-1 [GLP-1] receptor agonists, dipeptidyl peptidase-4 [DPP-4] inhibitors or pramlintide) to their treatment.

Participants measured and recorded plasma glucose during the trial using a single type of protocol-defined blood glucose meter supplied by the study sponsor, along with instructions for self-measurement of fasting glucose, at local investigators’ discretion. Glycaemic management was guided by review of self-measured plasma glucose (SMPG) values and HbA_1c_ levels for the individual participants.

Participants provided written consent, which was approved according to local regulations by appropriate health authorities, and by an independent ethics committee/institutional review board. The trial was conducted in accordance with the Declaration of Helsinki, ICH Good Clinical Practice and FDA 21 CFR 312.120 in the USA.

### Outcomes

In this post hoc analysis, we explored the possibility of annual rate of NSHEs being associated with time to first severe hypoglycaemic episode, time to first MACE, time to cardiovascular death and time to all-cause mortality. An NSHE was defined as SMPG <3.1 mmol/l (56 mg/dl), and severe hypoglycaemia defined as an episode requiring assistance of another person to actively administer carbohydrate or glucagon, or to take other corrective action [6]. MACE was a composite of cardiovascular death, non-fatal myocardial infarction and non-fatal stroke (three-point MACE), all of which were adjudicated in an independent and blinded manner by external event-adjudication committees. During the total trial period of 35,563 patient years of observation (median follow-up of 3.8 years), 27,933 NSHEs were registered. There were 433 severe hypoglycaemic episodes, 1302 first cases of three-point MACE, 497 cases of cardiovascular death and 828 cases of all-cause mortality [28].

### Statistical analyses

Data were analysed using a Cox proportional hazards model and the annual rate of NSHEs was modelled in a similar way to a previous study [26], as a time-dependent covariate with three levels: Group A: <2 NSHEs per year (reference group); Group B: 2–11 NSHEs per year; Group C: ≥12 NSHEs per year. All analyses consider time to first event.

The reference group comprised individuals with non-frequent, non-severe hypoglycaemia (defined as <2 episodes per year), and those with no reported NSHEs, based on the assumption that these two groups of patients could be combined.

Associations between annualised NSHEs and time to first event were analysed using Cox proportional hazards regression with minor hypoglycaemia rate. Minor hypoglycaemia refers to any NSHE (symptomatic or asymptomatic) with an SMPG measurement <3.1 mmol/l (56 mg/dl) as a time-dependent covariate and using a fixed-window approach where follow-up time was divided into 100-day windows [30, 31]. Analyses were made using an unadjusted model, and two models that adjusted for baseline characteristics. The first adjusted model accounted for age, sex, baseline HbA_1c_ and diabetes duration (≥15 years, yes/no). The second adjusted model accounted for renal function in addition to the other characteristics described above, classifying participants according to eGFR as normal (≥90 ml/min), mild impairment (<90–60 ml/min), moderate impairment (<60–30 ml/min) or severe impairment (<30 ml/min). HRs and 95% CIs for the associations of minor hypoglycaemia rate and time to first event were reported.

Three major sensitivity analyses were performed. The first was similar to the main analysis, except that the time-dependent covariate was updated each time a new severe hypoglycaemia event, MACE, cardiovascular death or overall death occurred, depending on the specific analysis, and was not baseline-adjusted. In the second sensitivity analysis, the first year of follow-up was used to classify participants according to their minor hypoglycaemia rate, and time to first event in the analysis was based on the remaining follow-up time (>1 year). Individuals with less than 1 year of follow-up were excluded from this analysis. Finally, in a third sensitivity analysis on MACE and cardiovascular death, all participants who had one or more severe hypoglycaemic episodes during follow-up were excluded from the analysis.

Finally, the data were analysed adjusted for insulin use (baseline or during trial, yes/no), and then analysed using a more stringent NSHE reference category of zero NSHE events: Group A: no NSHEs; Group B: >0 to <12 NSHEs; Group C: ≥12 NSHEs.

## Results

### Baseline characteristics

Table 1 shows the baseline characteristics of participants categorised according to their highest observed annual NSHE rate (Groups A–C). Baseline characteristics were similar for age, BMI and sex distribution. Participants in Group C (≥12 NSHEs per year) had a longer duration of diabetes and were less likely to be insulin naive.

**Table 1:** Baseline characteristics for the overall LEADER trial population and stratified by the annual rate of NSHEs

Variable	Overall (*N* = 9340)	Group A (<2 NSHEs per year) (*n* = 7019)	Group B (2–11 NSHEs per year) (*n* = 1919)	Group C (≥12 NSHEs per year) (*n* = 395)
Age (years)	64.3 (7.2)	64.2 (7.3)	64.6 (7.1)	64.2 (6.9)
Female (%)	35.7	35.3	36.1	40.8
BMI (kg/m^2^)	32.5 (6.3)	32.8 (6.4)	31.8 (6.0)	31.2 (6.2)
HbA_1c_ (mmol/mol)	71.5 (16.7)	71.8 (17.0)	70.8 (15.8)	70.1 (15.5)
HbA_1c_ (%)	8.7 (1.5)	8.7 (1.6)	8.6 (1.4)	8.6 (1.4)
Diabetes duration (years)	12.8 (8.0)	12.0 (7.7)	14.8 (8.2)	17.4 (8.8)
Insulin naive (%)	55.4	60.3	42.5	30.9
eGFR (ml min^−1^ [1.73 m]^−2^)	81.5 (27.7)	83.3 (27.6)	76.7 (27.0)	72.93 (26.6)
Chronic kidney failure (%)^a^	24.7	22.5	30.4	36.5
Existing CVD (%)	81.3	80.7	82.7	85.3
Systolic BP (mmHg)	135.9 (17.7)	136.1 (17.6)	134.9 (17.8)	136.5 (19.8)
Diastolic BP (mmHg)	77.1 (10.2)	77.5 (10.1)	75.9 (10.6)	75.8 (10.3)

Data are mean (SD) or %

Participants were grouped according to their highest annual NSHE rate. Owing to incomplete baseline information, seven individuals were not included

^a^eGFR <60 ml min^−1^ [1.73 m]^−2^

### Relationship between NSHEs and severe hypoglycaemia

For the three categories of NSHE rate, Group A (<2 NSHEs per year), Group B (2–11 NSHEs per year) and Group C (≥12 NSHEs per year), the numbers of severe hypoglycaemic episodes were 208, 46 and 13, respectively.

**Fig. 1:**
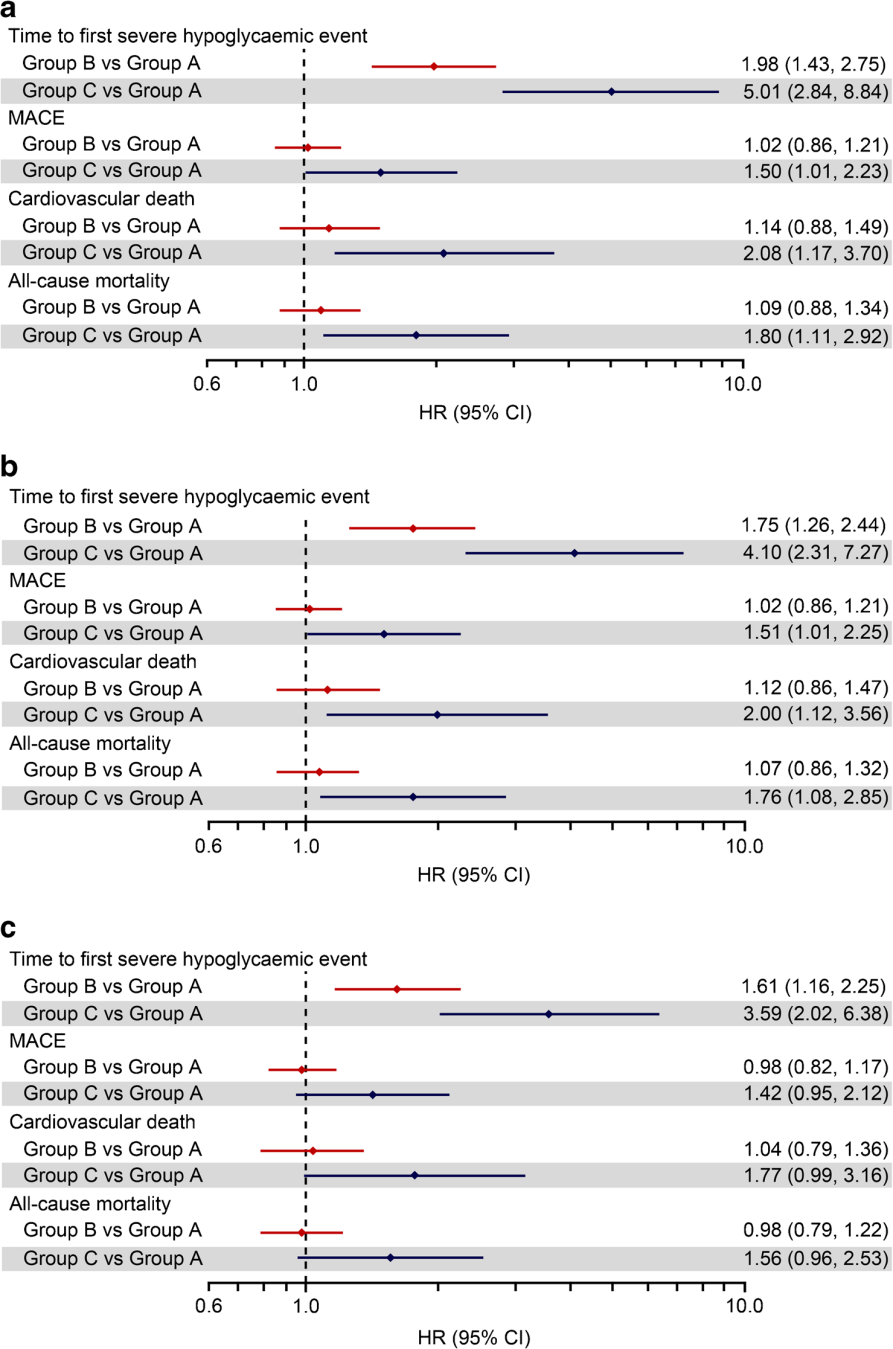
Time-dependent covariate analysis of a Cox proportional hazards model for severe hypoglycaemia, MACE, cardiovascular death and all-cause mortality by NSHE rate groups: (**a**) unadjusted; (**b**) adjusted for baseline characteristics (excluding renal function); (**c**) adjusted for baseline characteristics (including renal function). Reference group, Group A: <2 NSHEs per year; Group B: 2–11 NSHEs per year; Group C: ≥12 NSHEs per year

The main analysis showed that participants in both Group B and Group C experienced higher rates of severe hypoglycaemia compared with Group A (unadjusted HR 1.98 [95% CI 1.43, 2.75] and 5.01 [95% CI 2.84, 8.84], respectively; Fig. 1a). Results were similar when the main analysis was adjusted for baseline characteristics including renal function (Fig. 1b,c).

**Fig. 2:**
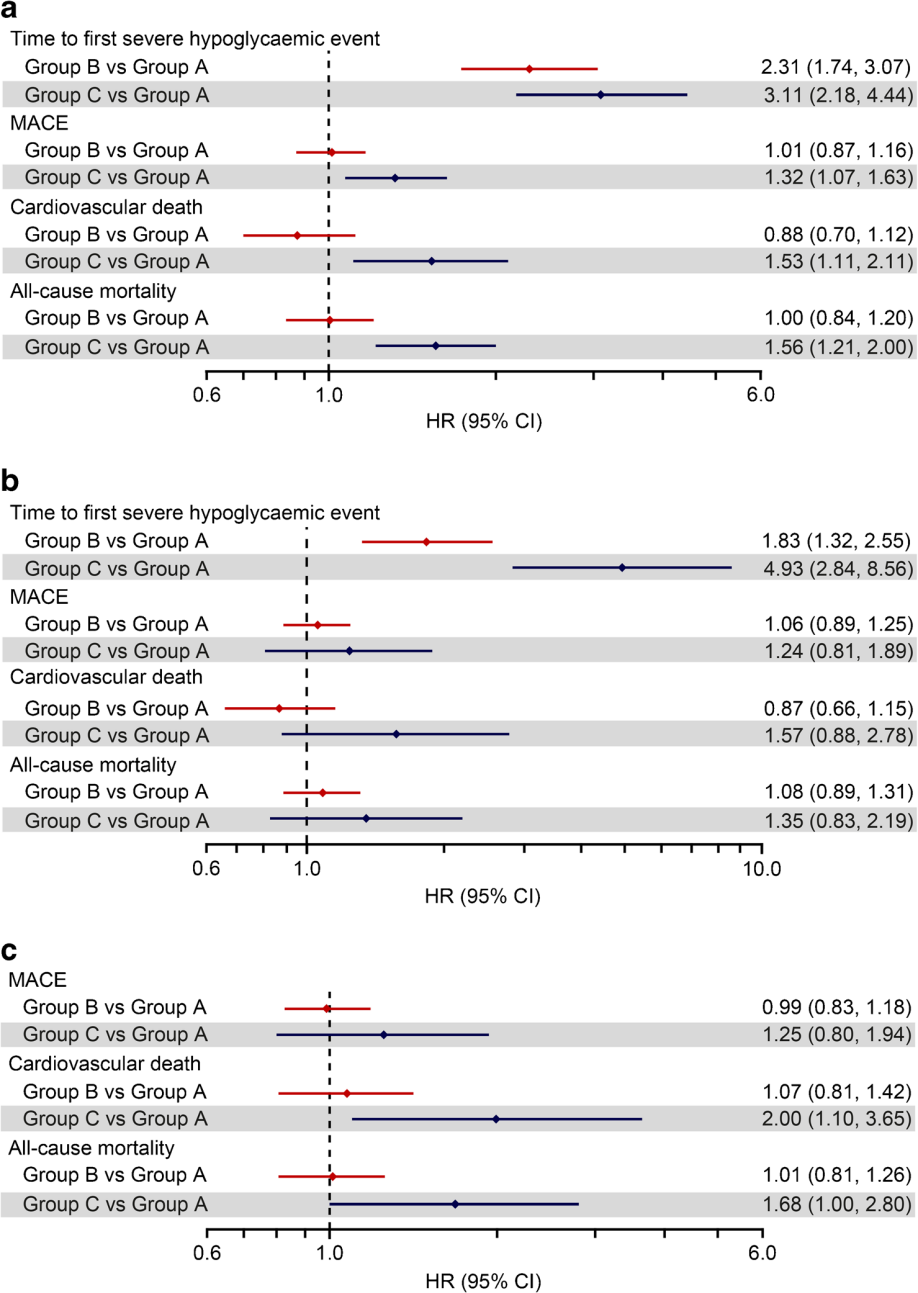
Sensitivity analyses for the association of NSHE and other events. (**a**) Time-dependent covariate sensitivity analysis of NSHEs and outcomes, where exposure time was split when a new outcome event occurred. (**b**) The first year of exposure was used to group participants in one of three risk categories, and the remainder of the exposure time was used to study the association between risk category and time to first event. (**c**) Same as main analysis but severe hypoglycaemia events were excluded from the dataset. Reference group, Group A: <2 NSHEs per year; Group B: 2–11 NSHEs per year; Group C: ≥12 NSHEs per year

In the first sensitivity analysis, where the time-dependent covariate was updated at each NSHE time, the HR for severe hypoglycaemia in Group C vs Group A was smaller compared with the main analysis, but the 95% CI was narrower (HR 3.11 [95% CI 2.18, 4.44]; Fig. 2a). The HR for Group B vs Group A in this sensitivity analysis was marginally higher than that observed in the main analysis (HR 2.31 [95% CI 1.74, 3.07]; Fig. 2a).

The second sensitivity analysis, which categorised participants by NSHE annual event rate in the first 12 months, was similar to the main analysis (HR 1.83 [95% CI 1.32, 2.55] and HR 4.93 [95% CI 2.84, 8.56]; Fig. 2b) for Group B vs A and Group C vs A, respectively.

When the data were adjusted for insulin use (baseline or during trial, yes/no), although point estimates were slightly attenuated, overall results were similar to the main analysis. HRs [95% CI] for Groups B and C, vs A, for severe hypoglycaemia were 1.72 [1.24, 2.39] and 4.09 [2.31, 7.22], respectively.

A repeat of the main analysis using a more stringent NSHE reference category (Group A: no NSHEs; Group B: >0 to <12 NSHEs; Group C: ≥12 NSHEs) did not change the results substantially. The unadjusted HRs for severe hypoglycaemia for Groups B and C vs Group A were 2.22 [95% CI 1.69, 2.92] and 5.95 [95% CI 3.34, 10.62], respectively.

### Relationship between NSHEs and cardiovascular outcomes

For the three categories of NSHE rate, Group A (<2 NSHEs per year), Group B (2–11 NSHEs per year) and Group C (≥12 NSHEs per year), the numbers of cardiovascular events were:

- MACE: 1128, 149 and 25, respectively
- Cardiovascular death: 422, 63 and 12, respectively
- Overall death: 711, 100 and 17, respectively.

The main analysis found that the HRs for adverse cardiovascular outcomes was similar between Groups A and B (Fig. 1). However, the higher rate of NSHEs in Group C was associated with a higher rate of MACE (HR 1.50 [95% CI 1.01, 2.23]), cardiovascular death (HR 2.08 [95% CI 1.17, 3.70]) and all-cause mortality (HR 1.80 [95% CI 1.11, 2.92]) in the main analysis (Fig. 1a). When baseline characteristics were accounted for (excluding renal function), the rates of MACE, cardiovascular death and all-cause mortality were consistent with the unadjusted analysis (Fig. 1b). The analysis that accounted for baseline characteristics, including renal function, showed a smaller HR between NSHE Groups C and A for MACE (HR 1.42 [95% CI 0.95, 2.12]), cardiovascular death (HR 1.77 [95% CI 0.99, 3.16]) and all-cause mortality (HR 1.56 [95% CI 0.96, 2.53]; Fig. 1c) compared with the unadjusted analysis.

The first sensitivity analysis (Fig. 2a) was consistent with the main analysis in demonstrating an increased risk of MACE, cardiovascular death and all-cause mortality in NSHE Group C vs Group A. The first sensitivity analysis had narrower CIs than the main analysis, suggesting an association between previous NSHE and MACE (HR 1.32 [95% CI 1.07, 1.63]), cardiovascular death (HR 1.53 [95% CI 1.11, 2.11]) and all-cause mortality (HR 1.56 [95% CI 1.21, 2.00]).

In the second sensitivity analysis, which categorised participants by NSHE event rate in the first 12 months and only considered follow-up after the first year, the total number of events was notably reduced compared with the main analysis (Fig. 2b). Compared with the main analysis, this sensitivity analysis resulted in smaller HRs between Group C and Group A for MACE (HR 1.24 [95% CI 0.81, 1.89]), cardiovascular death (HR 1.57 [95% CI 0.88, 2.78]) and all-cause mortality (HR 1.35 [95% CI 0.83, 2.19]).

In the third sensitivity analysis, when we excluded participants who experienced one or more severe hypoglycaemic episodes during follow-up, the association between NSHEs and cardiovascular death or overall death in Group C (HR 2.00 [95% CI 1.10, 3.65]; HR 1.68 [95% CI 1.00, 2.80], respectively) remained similar to the main analysis. However, the HR between NSHE Groups C and A for MACE was smaller (HR 1.25 [95% CI 0.80, 1.94]; Fig. 2c) than that seen in the main analysis.

When the data were adjusted for insulin use (baseline or during trial, yes/no), although point estimates were slightly attenuated, overall results were similar to the main analysis. HRs [95% CI] for Group B and C, vs A, for MACE were 0.98 [0.83, 1.17] and 1.42 [0.95, 2.12]; for cardiovascular death were 1.13 [0.87, 1.48] and 2.04 [1.15, 3.64]; and for overall death were 1.05 [0.85, 1.30] and 1.72 [1.06, 2.79].

A repeat of the main analysis using a more stringent NSHE reference category (Group A: no NSHEs; Group B: >0 to <12 NSHEs; Group C: ≥12 NSHEs) did not change the results substantially. The HRs for MACE for Groups B and C vs Group A were 0.99 [95% CI 0.88, 1.12] and 1.49 [95% CI 1.00, 2.22], respectively. The HRs for cardiovascular death for Groups B and C vs Group A were 0.90 [95% CI 0.74, 1.09] and 1.97 [95% CI 1.11, 3.51], respectively. The HRs for overall death for Groups B and C vs Group A were 1.04 [95% CI 0.90, 1.20] and 1.81 [95% CI 1.11, 2.94], respectively.

## Discussion

This post hoc analysis of the LEADER data identified an association between NSHEs and severe hypoglycaemic episodes. Importantly, the study showed that a high rate of NSHEs (≥12 NSHEs per year) was also associated with higher rates of adverse cardiovascular outcomes, including MACE, cardiovascular death and all-cause mortality, in individuals with type 2 diabetes.

Our analysis confirms recent evidence associating NSHEs with increased risk of severe hypoglycaemia [26, 27]. Festa et al. conducted an analysis of patients with type 2 diabetes initiating insulin, investigating the relationship between NSHEs and severe hypoglycaemia using a clinical trial database (*N* = 2931 from three trials) [26]. They found that the risk of severe hypoglycaemia was higher for participants experiencing multiple NSHEs compared with those experiencing ≤1 NSHEs per month (HR 4.24 [95% CI 2.57, 6.99] *p* < 0.0001) [26]; our analysis results are consistent with these data. Roussel et al. [27] conducted a systematic analysis of published data for insulin-treated patients, and found that the incidence of NSHEs, especially nocturnal NSHEs, were the best predictor of severe hypoglycaemic events.

We also found an association between the higher rate of NSHEs (≥12 NSHEs per year) and adverse cardiovascular outcomes, consistent with multiple sensitivity analyses. There is considerable evidence demonstrating an association between hypoglycaemia and the development of cardiovascular complications in type 2 diabetes [32–34], including secondary analyses of a number of large diabetes trials such as ADVANCE (Action in Diabetes and Vascular Disease: Preterax and Diamicron MR Controlled Evaluation) [13] and LEADER [12]. It is worth noting that this association was not seen in the ORIGIN trial, perhaps because the NSHEs patient group in ORIGIN experienced a median of 0.34 NSHEs over a median of 6.2 years, compared with ≥12 NSHEs per year in Group C of this study [22]. However, parallels can be drawn between LEADER and ORIGIN [22]. While NSHEs were not associated with cardiovascular outcomes after adjustment in ORIGIN, in LEADER, no association was found between the rate of NSHEs and adverse cardiovascular outcomes in Group C when accounting for baseline characteristics, including renal function, or in Group B (2–11 NSHEs per year). Both studies found severe hypoglycaemia associated with a higher risk for cardiovascular outcomes.

Participants enrolled in the LEADER study who experienced severe hypoglycaemic episodes were more likely to experience MACE, cardiovascular death and all-cause mortality compared with those with no experience of severe hypoglycaemia. The risk of adverse cardiovascular events was particularly high in the immediate period following episodes of severe hypoglycaemic events [12]. As these findings are based on a post hoc analysis of LEADER, it remains uncertain if hypoglycaemia is a marker or mediator of associated cardiovascular risk. A bi-directional association between severe hypoglycaemia and cardiovascular events, with greater risk of cardiovascular events after severe hypoglycaemia and vice versa, particularly in individuals with comorbidities, is evident [18]. The presence of comorbidities does not, however, fully explain this association [35]. Evidence from the DEVOTE trial also supports an association between severe hypoglycaemia and MACE. While this analysis cannot be considered conclusive evidence of a causal link, the results imply that individuals with high rates of MACE are also those with high rates of hypoglycaemia. In the absence of an RCT to assess this relationship, which is neither possible nor ethical, evidence supports the hypothesis that hypoglycaemia is both a risk marker and risk factor for cardiovascular events [19]. Furthermore, independent of causality, reducing the risk of any hypoglycaemia by lifestyle intervention or pharmacological solutions will benefit individuals using insulin, including those at high cardiovascular risk.

Interestingly, when participants experiencing one or more severe hypoglycaemic episodes were excluded from the analysis, the association between NSHEs and MACE was reduced in those experiencing ≥12 NSHEs per year; however, the relationships between NSHEs and cardiovascular death, or NSHEs and overall death, remained consistent with the main analysis. This indicates that the association between NSHEs and MACE is at least partly driven by those individuals who have a high rate of NSHEs and who also have severe hypoglycaemic events. Yet even when all participants who have experienced an episode of severe hypoglycaemia have been removed from the dataset there is still an association between NSHEs and cardiovascular death. There are a number of pathways involved in the response to hypoglycaemia that may be causally linked to adverse cardiovascular outcomes, particularly if non-severe events are experienced repeatedly over a period of weeks or months. Experimentally induced moderate hypoglycaemia (defined as 2.5 mmol/l [45 mg/dl]) is associated with acute and persistent prothrombotic effects, illustrating a possible mechanism by which hypoglycaemia might increase cardiovascular risk [36]. Hypoglycaemia also provokes an increase in plasma aldosterone, and this may exacerbate vascular dysfunction through the activation of the mineralocorticoid receptor [37]. A proinflammatory response characterised by platelet and monocyte activation is initiated during experimentally induced acute moderate hypoglycaemia (blood glucose of approximately 2.5 mmol/l [45 mg/dl]), which could also play a role in the adverse cardiovascular outcomes reported [32, 38, 39]. Finally, in individuals with type 2 diabetes and at high cardiovascular risk, hypoglycaemia has also been associated with increased risk of cardiac arrhythmias, possibly via perturbations in cardiac autonomic tone and abnormal repolarisation [40, 41].

In LEADER, over 80% of people had prior CVD at randomisation. The current findings therefore mainly apply to a secondary prevention population, who are at high risk of recurrent cardiovascular events.

The association between NSHEs and adverse cardiovascular outcomes was attenuated when our analysis accounted for baseline renal function. This could be due to shared vulnerabilities in people with chronic kidney disease for hypoglycaemia and adverse cardiovascular events. A similar association has been shown in a subanalysis of DEVOTE, which found that after adjustment, participants with a baseline eGFR of 60–<90 ml min^−1^ [1.73 m]^−2^ no longer had significantly higher risks of cardiovascular death and all-cause mortality compared with those with baseline eGFR ≥90 ml min^−1^ [1.73 m]^−2^, in addition to a non-significant increased risk of severe hypoglycaemia [42].

Aside from an association between severe hypoglycaemia and cardiovascular outcomes, in the context of hypoglycaemia, an aspect of interest is ‘frailty’ [43]. Chronic inflammation and insulin resistance may increase frailty incidence, while a frail status may also cause these conditions, which in turn may be associated with CVD [43]. Frailty has been found to be an independent risk factor for CVD [44], and a significant relationship has been demonstrated between frailty and CVD [45]. Further, hypoglycaemia in older people is associated with significant morbidity, and recurrent hypoglycaemia is associated with deterioration in general health, which is likely to lead to frailty and poor outcomes [46]. This relationship between hypoglycaemia and frailty appears to be bi-directional, mediated through factors such as undernutrition and reduced muscle mass [46]. Thus, it could be argued that severe hypoglycaemia and cardiovascular outcomes are markers of a frail state in which the patient is more likely to have both severe hypoglycaemia and CVD, as well as other conditions, including non-cardiovascular death.

The main limitation of the study is that it was a post hoc hypothesis-generating analysis, rather than a primary analysis with a specific endpoint to investigate the relationship between NSHEs, severe hypoglycaemia and adverse cardiovascular outcomes. We were also unable to conduct an analysis adjusting for the duration of insulin as this information was not available for LEADER. Additionally, SMPG measurements were not embedded into the LEADER protocol, and were likely to be more common in those with prior glucose variability, previous experience of hypoglycaemia and those on insulin therapy. However, the large size of the population studied, the relatively high number of NSHEs available for the analysis, and the considerable length of follow-up provides a strong basis for the evidence of an association of NSHEs with severe hypoglycaemia and adverse cardiovascular outcomes.

In conclusion, the data from LEADER, a long-term cardiovascular outcomes trial, demonstrated that higher rates of NSHEs were associated with higher rates of severe hypoglycaemia, MACE, cardiovascular death and all-cause mortality in individuals with type 2 diabetes. We would argue strongly that, whatever the underlying driver of the link between CVD and hypoglycaemia, it is important that healthcare professionals and patients take steps to reduce this common complication of treatment.

## Data Availability

The patient-level analysis datasets for the research presented in the publication are available from the corresponding author on reasonable request.

## Abbreviations

ACCORD: Action to Control Cardiovascular Risk in Diabetes
LEADER: Liraglutide Effect and Action in Diabetes: Evaluation of Cardiovascular Outcome Results
MACE: Major adverse cardiovascular event
NSHE: Non-severe hypoglycaemic episode
ORIGIN: Outcome Reduction With Initial Glargine Intervention
SMPG: Self-measured plasma glucose
